# PTH1R Suppressed Apoptosis of Mesenchymal Progenitors in Mandibular Growth

**DOI:** 10.3390/ijms252312607

**Published:** 2024-11-24

**Authors:** Chen Cui, Chuang Lu, Yanling Cai, Yuhua Xiong, Yihong Duan, Kaiwen Lan, Yi Fan, Xuedong Zhou, Xi Wei

**Affiliations:** 1 Hospital of Stomatology, Guanghua School of Stomatology, Sun Yat-sen University, Guangzhou 510055, China; cuich7@mail.sysu.edu.cn (C.C.); (luch8@mail2.sysu.edu.cn (C.L.); caiyling3@mail.sysu.edu.cn (Y.C.); xiongyh8@mail2.sysu.edu.cn (Y.X.); duanyh6@mail2.sysu.edu.cn (Y.D.); lankw@mail2.sysu.edu.cn (K.L.); 2 Guangdong Provincial Key Laboratory of Stomatology, Guangzhou 510055, China; 3 State Key Laboratory of Oral Diseases & National Clinical Research Center for Oral Diseases, West China Hospital of Stomatology, Sichuan University, Chengdu 610041, China; yifan@scu.edu.cn

**Keywords:** PTH1R, stem cells, bone remodeling/regeneration, receptors, cell signaling, apoptosis, craniofacial biology/genetics

## Abstract

Genetic abnormalities of the parathyroid hormone 1 receptor (PTH1R) lead to profound craniomaxillofacial bone and dentition defects on account of inappropriate tissue metabolism and cellular differentiation. The coordinated activity of differentiation and viability in bone cells is indispensable for bone metabolism. Recent research demonstrates mesenchymal progenitors are responsive to PTH1R signaling for osteogenic differentiation, whereas the effect of PTH1R on cellular survival remains incompletely understood. Here, we report that mice with deletion of PTH1R in Prx1-positive mesenchymal cells (*Prx1Cre;PTH1R^fl/fl^*) exhibit decreased alveolar bone mass due in part to apoptotic response activation. The exploration of oral bone-derived mesenchymal stem cells (OMSCs) with PTH1R deficiency suggests PTH1R signaling modulates OMSCs’ apoptosis by interfering mitochondrial function and morphology. The underlying molecular mechanisms are studied by transcriptome sequencing analysis, finding that inositol trisphosphate receptor-3 (IP3R-3), an endoplasmic reticulum calcium channel protein, serves as a modulator of pro-apoptosis in OMSCs. Furthermore, we find PTH1R and its downstream protein kinase A (PKA) pathway dampen IP3R-3’s expression. Of note, OMSCs with IP3R-3 overexpression recapitulate the PTH1R-deletion phenotypes, while IP3R-3 silence rescues mitochondrial dysfunction. Altogether, our study uncovers the anti-apoptotic function of PTH1R signaling in OMSCs and proves that excess apoptosis partly contributes to a weakening potential of osteogenic differentiation and aberrant mandibular development.

## 1. Introduction

Bone remodeling and self-repairing are necessary for the mandible because it suffers from a sustained biting force [1]. Different from the main ossification fashion in the skeleton, the majority of the mandible (excluding condylar neck and upper ramus) develops and repairs through intramembranous ossification [2]. During this process, neural crest-derived mesenchymal cells proliferate and some of them change their biological traits [3]. Orofacial bone marrow-derived mesenchymal stem cells (OMSCs) are one of major subtypes and can differentiate into osteolineage cells [4]. OMSCs and their postnatal progeny are present throughout life and tightly regulate osteogenesis during the modeling and remodeling processes in the mandible [5]. They go through proliferation, matrix maturation, and mineralization, and the homeostasis of osteogenic differentiation and cellular viability are critical for skeletal integrity [6]. Apoptosis occurs throughout all stages, and apoptotic mesenchymal cells are necessary for bone metabolism, supported by the observation that they are near the osteogenic front of developing bone and regenerative sites [7]. However, inappropriate apoptosis disrupts the balance between the survival and death of bone cells, contributing to diseases, including periodontal disease, glucocorticoid-induced osteonecrosis, and degenerative joint diseases [8].

Parathyroid hormone 1 receptor (PTH1R), a G-protein-coupled receptor activated by parathyroid hormone (PTH) and its related protein (PTHrP), is extensively expressed in diverse tissues. During bone and cartilage development, activation of PTH1R signaling is indispensable for bone formation by directing mesenchymal cellular fate, promoting osteogenesis, and maintaining cellular survival [9]. Genetic mutations of PTH1R are associated with multiple skeletal defects. Jansen’s metaphyseal chondrodysplasia, a disease caused by constitutively active mutant of PTH1R, leads to short limbed dwarfism, and some studies believe it is due to retarded chondrocyte differentiation and decreased chondrocyte apoptosis in tibial growth plate [10]. In transgenic mouse model with constitutive activation of PTH1R, authors observed accelerated osteoblastic proliferation and decreased apoptosis in trabecular bone [11]. On the other hand, mice with PTH1R deficiency in Lepr^+^ cell lineage exhibited a restrain number of stem cells in alveolar bone marrow [12]. In dental follicle, PTH1R deletion in Sp7^+^ or PTHrP^+^ progenitor cells dampen cellular proliferation [13,14]. Those findings point out PTH1R signaling participates in cellular survival and subsequent bone formation in appendix skeleton and craniofacial region. Yet, the effect of PTH1R signaling on cellular apoptosis is still controversial. One of the clues come from a bi-directional effect of PTH on odontoblasts, which means the duration of PTH exposure may be an influential factor in anti- or pro-apoptotic effects via PTH1R/PKA or PKC signaling, respectively [15]. In the skeleton, PTH1R signaling suppresses apoptosis of mature osteoblasts and osteocytes, but activates apoptosis of hypertrophic chondrocytes. Thus, understanding the impact of PTH1R on OMSCs’ apoptosis will give new insights into the mechanism of PTH1R signaling in mandibular development.

Mitochondria play a core role in apoptotic process, which is induced by calcium ion (Ca^2+^) overload and reactive oxygen species (ROS) accumulation. Ca^2+^ acts as a ubiquitous second messenger in many intracellular biological processes and effects proliferation, differentiation and apoptosis of osteolineage cells [16]. This modulation is highly dependent on a variety of Ca^2+^ flux patterns, which are realized by the Ca^2+^ transfer in cytosolic Ca^2+^, intracellular Ca^2+^ storages and extracellular Ca^2+^ pools [17]. Endoplasmic reticulum (ER) is the largest store of intracellular Ca^2+^, and Ca^2+^ release from ER relies on Ca^2+^ channels including inositol trisphosphate receptors (IP3Rs) and ryanodine receptors (RyRs) [18,19]. IP3R-3 is mainly responsible for Ca^2+^ release from ER to mitochondria and has been regarded as tumorigenesis target to modulate cellular proliferation and apoptosis. Proper Ca^2+^ release via IP3R-3 promotes mitochondrial ATP production, but mitochondrial Ca^2+^overload results in Ca^2+^-dependent apoptosis [18,20]. However, the effect of IP3R-3 in OMSCs remains unknown.

Herein, we asked what role IP3R-3 plays in PTH1R signaling during mandibular bone formation. In our previous study, we generated a type of transgenic mice that conditionally ablated PTH1R in pair-related homeobox gene (Prx1) positive-MSCs, and found mice with the PTH1R mutation exhibited delayed tooth eruption, possibly due to reduced alveolar bone formation [21]. Subsequently, we found that PTH1R deletion limits bone mass, partly due to an apoptotic response activation of OMSCs. Notably, the loss of PTH1R in OMSCs interferes with mitochondrial function and morphology, with IP3R-3 being a potential modulator. Our findings reveal novel insights into PTH1R signaling in mineralization and the viability of OMSCs.

## 2. Results

### 2.1. Conditional Knockout PTH1R Induced Downregulation of Alveolar Bone Mass

**Figure 1:**
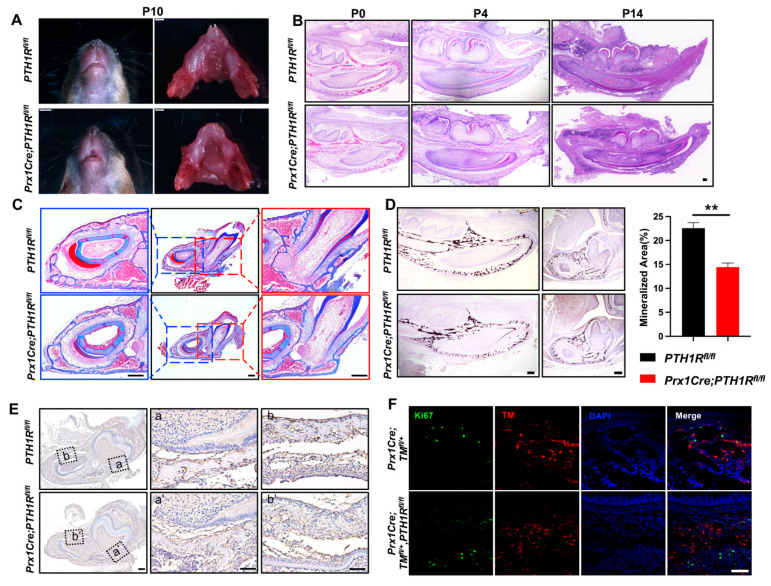
PTH1R deficiency in Prx1^+^-mesenchymal cells causes tooth eruption delay based on downregulation of bone mass. (**A**) Gross phenotype of the mandible at postnatal days 10 (P10) in *PTH1R^fl/fl^* and *Prx1Cre*;*PTH1R^fl/fl^* mice. n = 3. (**B**) H&E staining of mandible at P0, P4, P14. n = 3–4. Scale bar: 200 μm. (**C**) Masson’s trichrome staining in alveolar bone at P14. n = 3. Scale bar: 200 μm. (**D**) Representative images of Von Kossa staining and quantitative analyses. Sagittal planes (left, n = 3) and coronal (right, n = 4). Scale bar: 200 μm. (**E**) IHC staining of cathepsin K showed the distribution of osteoclasts. (a, a’) are shown at higher magnification at the base of molar and (b, b’) are shown at higher magnification surrounding incisor. n = 3. Scale bar: 200 μm. (**F**) IF staining of Ki67 in mandibular marrow. Ki67^+^ cells (Green) and Prx1^+^ cells (Red). n = 3. Scale bar: 50 μm. ** *p* < 0.01 was considered as a statistically significant difference between *PTH1R^fl/fl^* and *Prx1Cre*;*PTH1R^fl/fl^*. All data are shown as the mean ± SEM.

We first generated *Prx1Cre;PTH1R^fl/fl^* (mutant) mice in which PTH1R was specifically ablated by the expression of Cre recombinase under the *Prx1* promoter control [22], and found an arrested mandibular incisor eruption in mutants at postnatal day 10 (P10) (Figure 1A). In line with our previous reports, we believe the PTH1R deficiency cause a phenotype of tooth eruption delay due to the loss of eruption force from alveolar bone, according to micro-computed tomography and immunostaining of osteogenic regulators [21]. Subsequently, H&E staining was performed, exhibiting the histology of mandibles at P0, P4, and P14. We observed a significant incisor eruption delay in mutant mice without tooth structure abnormality (Figure 1B). Moreover, Masson’s trichrome staining and Von Kossa staining further confirmed the limited mineralized area surrounding incisors and at the base of molars in mutant mice (Figure 1C,D). Combined with our previous finding that alveolar bone marrow surrounding incisors and molars is the main place of Prx1^+^-MSCs distribution [21], these data suggest that PTH1R-ablation in Prx1^+^-MSCs directly cause alveolar bone volume reduction.

We have reported the bone formation effect in PTH1R mutants, but bone mass is the coordinated result of bone formation and resorption. So, we then evaluated bone resorption by the number of osteoclasts. IHC staining marked osteoclasts by cathepsin K, showing a similar distribution of osteoclasts in mutant and wild-type mice (Figure 1E), which repeatedly imply that bone formation may occupy a more critical role. However, though research has proven the well-established mechanism that PTH1R ablation hinders osteogenic differentiation of MSCs and bone formation, it cannot explain our observation of a decreased number of Prx1^+^-MSCs in mutant mice [21]. The orchestration between cellular proliferation and death in bone marrow directly effects the quantity and quality of osteoblastic lineage cells. So, we subsequently detected proliferous Prx1^+^-cells in mutant mice with an R26R-tomato reporter system (*Prx1Cre;TM^fl/+^*,*PTH1R^fl/fl^*), in which Prx1^+^-cells were labeled by red fluorescence. We sought to count the number of double-positive cells in IF staining, but the result showed that there was no difference when PTH1R was deleted in Prx1^+^-cells (Figure 1F). Therefore, we hypothesize that cellular death may be core in reducing bone mass.

### 2.2. PTH1R-Ablation Promoted Apoptosis of Mesenchymal Cells Both In Vivo and In Vitro

**Figure 2:**
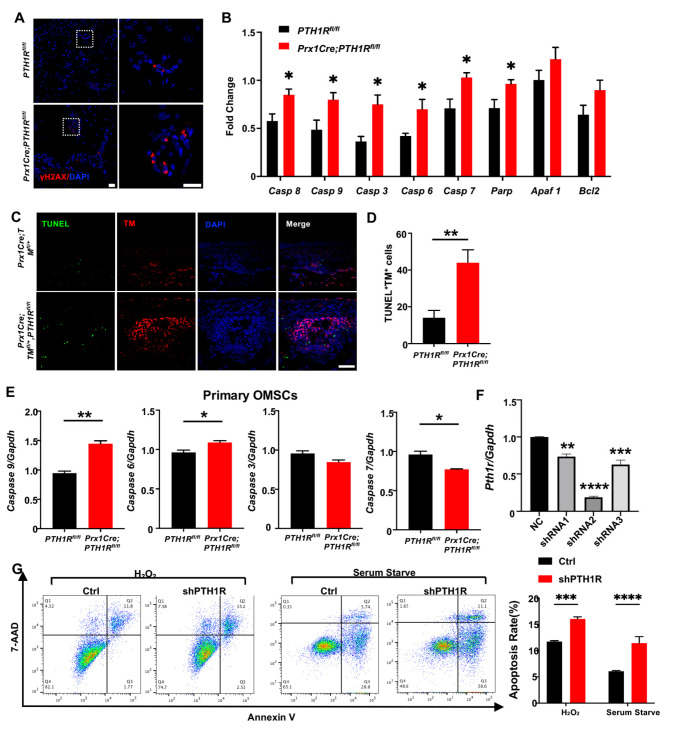
PTH1R deletion contributed to apoptotic response activation in mesenchymal cells. (**A**) IF staining of γH2AX in mandibular marrow exhibited an increased DNA damage in mutant. n = 3. Scale bar: 20 μm (left), 10 μm (right). (**B**) Apoptotic-related gene expression in mandibular tissue. n = 3. (**C**,**D**) Representative pictures of TUNEL staining and quantitative counting exhibit the number of double-positive cells (apoptotic Prx1^+^-cells) in alveolar bone marrow. n = 3. Scale bar: 50 μm. (**E**) Genes expression of *Casp 6*, *Casp 7*, *Casp 9* and *Casp 3* in primary OMSCs. n = 3. (**F**) Gene expression of PTH1R when OMSCs were transfected with three different shRNAs (shRNA1–3). n = 3. (**G**) Annexin V/7-AAD detected an apoptotic population in PTH1R-ablated OMSCs (shPTH1R) and negative control group (Ctrl) after OMSCs were treated with H_2_O_2_ for 24 h or in serum free medium for 72 h. n = 4. * *p* < 0.05, ** *p* < 0.01, *** *p* < 0.001, **** *p* < 0.0001 was considered as a statistically significant difference between *PTH1R^fl/fl^* and *Prx1Cre*;*PTH1R^fl/fl^* (**B**,**D**,**E**) or different group versus NC (**F**) or Ctrl and shPTH1R (**G**). All data are shown as the mean ± SEM.

Then, IF staining was performed to observe DNA damage in mandible by its biomarker, histone H2A.X phosphorylation (γH2AX), exhibiting a high level of fluorescence intensity in bone marrow cells of mutant mice (Figure 2A). Subsequently, the transcription levels of apoptosis-related markers, including caspase cascade genes (*Caspase 8*, *9*, *3*, *6*, and *7*) and *poly ADP-ribose polymerase* (*PARP*), were detected and augmented in the mandibular tissue of mutant mice (Figure 2B). TUNEL assay showed the distribution of apoptotic cells in alveolar bone marrow. TUNEL-positive apoptotic cells were significantly gathered in mutant mice, and the number of apoptotic PTH1R-ablated cells (double-positive cells) was significantly up-regulated (Figure 2C,D). These results implied that the booming apoptosis in bone marrow stromal cells is the possible reason for the restrained number of Prx1^+^-MSCs.

Next, we cultured OMSCs derived from alveolar bone marrow of *Prx1Cre;PTH1R^fl/fl^* and *PTH1R^fl/fl^* mice. In line with the in vivo observations, primary OMSCs from mutant mice showed high expression of initiator caspase (*Casp 9*) and effector caspase (*Casp 6*), implying a possible active progress of apoptosis (Figure 2E). However, other apoptosis-related markers have no similar results, possibly because Prx1^+^-MSCs only occupied a part of OMSCs. To efficiently ablate PTH1R in cultured cells, OMSCs were transfected with three different shRNAs, and shRNA2 showed a high-efficient silencing of PTH1R expression (Figure 2F), thus shRNA2 was performed against PTH1R in this study (shPTH1R). Flow cytometry assays further showed a significant increase of the apoptotic population among shPTH1R OMSCs during apoptosis induction (Figure 2G). Those findings further prove that PTH1R enhances anti-apoptotic ability of OMSCs.

### 2.3. PTH1R Deficiency Promoted Mitochondrial Associated Apoptosis in OMSCs

**Figure 3:**
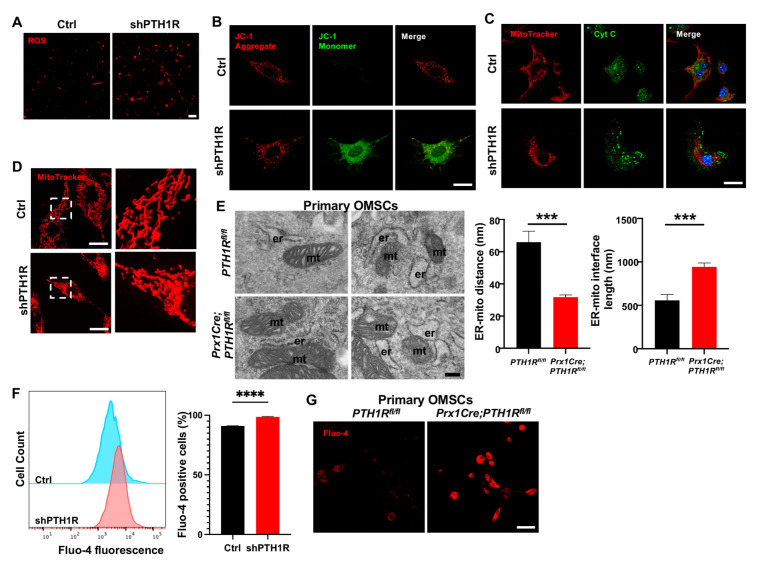
PTH1R signaling in OMSCs suppresses the mitochondrial-associated apoptotic process. (**A**) ROS levels in living OMSCs were detected with a cell-permeable fluorogenic probe (red). n = 3. Scale bar: 100 μm. (**B**) Representative image of JC-1 staining in Ctrl and shPTH1R groups. n = 6. Scale bar: 25 μm. (**C**) Double fluorescence staining of cytochrome c (Cyt C) and MitoTracker showing the intracellular distribution of Cyt C. n = 5. Scale bar: 25 μm. (**D**) MitoTracker staining in alive cells. Higher magnifications of boxed areas exhibited more fragmented mitochondria in PTH1R-deleted OMSCs. n = 3. Scale bar: 25 μm. (**E**) TEM observed the ER-mitochondrial ultrastructure in two groups, and the average distance and interface length (with 100-nm gap distance) were analyzed. n = 6. Scale bar: 200 nm. (**F**) Flow cytometry assay of Fluo 4-AM Ca^2+^ indicator detected intracellular Ca^2+^ concentration in shPTH1R and its control. n = 4. (**G**) Fluo 4-AM labeling Ca^2+^ in single-cell suspensions from mandible marrow tissue. n = 3. Scale bar: 50 μm. *** *p* < 0.001, **** *p* < 0.0001 was considered as a statistically significant difference between *PTH1R^fl/fl^* and *Prx1Cre*;*PTH1R^fl/fl^* (**E**) or Ctrl and shPTH1R (**F**). All data are shown as the mean ± SEM.

Then, we investigated the effects of diminished PTH1R in subcellular structure and function. ROS assay revealed an accumulation of intracellular ROS in shPTH1R (Figure 3A). JC-1 staining detected a stronger green fluorescence during PTH1R knockdown, suggesting an inchoate stage of apoptosis (Figure 3B). Mitochondrial dysfunction would lead to the release of small pro-apoptotic molecules, such as cytochrome c (Cyt C). Immunocytochemistry (ICC) staining of Cyt C (green) and MitoTracker (red) were performed, and revealed less overlap in shPTH1R, indicating an increased release of Cyt C from the mitochondria into the cytoplasm (Figure 3C). Interestingly, higher magnifications of MitoTracker staining exhibited the fragmented subtype (short rods or spheres) of mitochondria in shPTH1R, which means that PTH1R signaling impacts both mitochondrial function and morphology (Figure 3D).

Furthermore, single-OMSC suspensions from alveolar bone marrow of mutant mice were detected by TEM to elucidate mitochondrial structure, finding a closer spatial relationship between ER and mitochondria (Figure 3E). Since Ca^2+^ participated in the communication between ER and mitochondria, we used the fluorescent Fluo 4-AM Ca^2+^ indicator to identify whether PTH1R deficiency interfered with level of intracellular Ca^2+^. As shown in Figure 3F,G, PTH1R knockdown significantly increased basal intracellular Ca^2+^ in both shPTH1R and primary OMSCs from mutant mice. Collectively, these findings imply that PTH1R-deficiented OMSCs is associated with aberrant subcellular structures and cytoplasmic components.

### 2.4. IP3R-3 Was Highly Expressed in PTH1R-Mutant Mice and OMSCs

**Figure 4:**
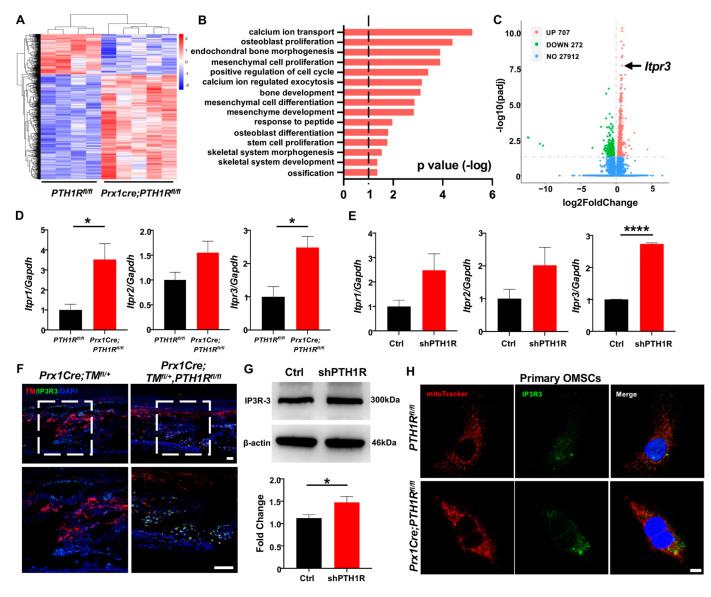
PTH1R deficiency enhances the transcription and translation levels of IP3R-3. (**A**) Heatmap of differentially expressed genes (DEGs) in mandible from *Prx1Cre*;*PTH1R^fl/fl^* and *PTH1R^fl/fl^* mice at P10. Red indicates upregulated expression, and blue indicates downregulated expression. n = 4 in *PTH1R^fl/fl^* and n = 5 in *Prx1Cre*;*PTH1R^fl/fl^*. (**B**) Gene ontology (GO) enrichment analysis of DEGs. (**C**) Volcano plot of DEGs showing significant highly expression of *Itpr3* when PTH1R ablation. (**D**) Gene expression of IP3Rs (*Itpr1*, *Itpr2* and *Itpr3*) in mandible. n = 3–4. (**E**) Gene expression of IP3Rs in PTH1R-ablated OMSCs. n = 3. (**F**) IF staining showing the expression of IP3R-3 (Green) in Prx1^+^ cells (Red) in mandible. Higher magnifications of boxed areas exhibited increased IP3R-3^+^ cells distribution at alveolar bone marrow. n = 3 in control mice and n = 4 in mutant mice. Scale bar: 50 μm. (**G**) Western blot identified the higher translation level of IP3R-3 in PTH1R deleted OMSCs. n = 3. (**H**) ICC staining of IP3R-3 and MitoTracker analyzed the distribution of IP3R-3 in primary OMSCs. n = 3. Scale bar: 25 μm. * *p* < 0.05, **** *p* < 0.0001 was considered as a statistically significant difference between *PTH1R^fl/fl^* and *Prx1Cre*;*PTH1R^fl/fl^* (**D**) or Ctrl and shPTH1R (**E**,**G**). All data are shown as the mean ± SEM.

In order to investigate the major regulator for Ca^2+^ uptake in mitochondria, we isolated mRNA from mandibles of *Prx1Cre;PTH1R^fl/fl^* and *PTH1R^fl/fl^* mice and performed transcriptome sequencing. Differentially expressed genes (DEGs) were obtained and exhibited by a heatmap (Figure 4A). Gene ontology (GO) analysis showed that the DEGs were associated with regulation of skeletal system morphogenesis, cellular proliferation and differentiation, and calcium ion transport (Figure 4B). Among a total of 28,891 genes expressed, 707 genes were significantly upregulated in *Prx1Cre;PTH1R^fl/fl^* mice. It is noteworthy that *Itpr3*, an encoding gene of IP3R-3 protein, was highly expressed in PTH1R-mutant mice (Figure 4C).

The IP3Rs family is the predominant protein group of Ca^2+^ release channels on ER, and there are three isoforms (IP3R-1, IP3R-2 and IP3R-3), which are respectively encoded by *Itpr1*, *Itpr2*, and *Itpr3*. We first tested the expression of three subtypes when PTH1R deletion. Performing qRT-PCR analysis, we found that although the three subtypes have enhanced tendencies in both mandibular tissue and OMSCs, only the expression of IP3R-3 has a stable statistic difference (Figure 4D,E). Moreover, the distribution of IP3R-3 was observed by IF staining. The results showed a higher amount of Prx1^+^-cells expressing IP3R-3 in the alveolar bone marrow of mutant mice (Figure 4F). Likewise, western blot showed that the protein level of IP3R-3 was higher in shPTH1R, which is in line with the sequencing analysis (Figure 4G). Furthermore, ICC staining confirmed a rising overlap area of IP3R-3 (green) and MitoTracker (red) in PTH1R-deleted primary OMSCs, implying a close relationship between IP3R-3 and mitochondria (Figure 4H). In our current analysis, we suspected the pivotal role of IP3R-3 in aberrant subcellular structures during PTH1R knockout.

### 2.5. PTH1R Negatively Regulated IP3R-3 Expression via PKA Signaling

**Figure 5:**
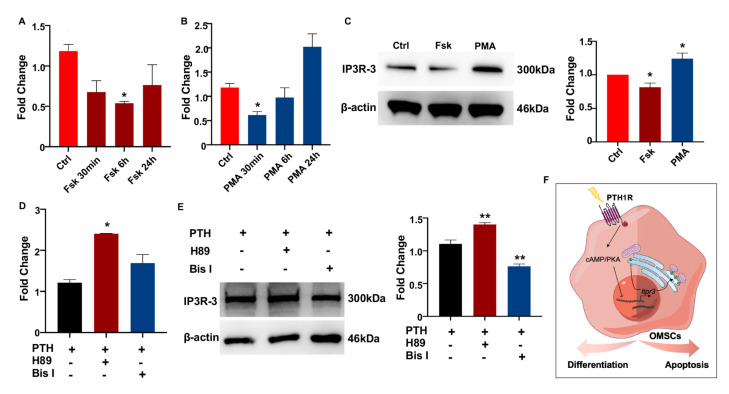
PTH1R signaling inhibits the expression of IP3R-3 by PKA pathway. (**A**,**B**) Gene expression of IP3R-3 when PKA activator (Fsk) and PKC activator (PMA) treatment. n = 3. (**C**) Western blot identified the translation of IP3R-3 after 24 h of Fsk and PMA induced. n = 3. (**D**,**E**) The transcription and translation levels of IP3R-3 in OMSCs with 2 h of PKA inhibitor (H89) or PKC inhibitor (Bis I) treatment prior to 10 min of PTH exposure. n = 3. (**F**) Schematic summary diagram showing the regulation of PTH1R in differentiation and survival of OMSCs. * *p* < 0.05, ** *p* < 0.01 was considered as a statistically significant difference versus Ctrl (**A**–**C**) or PTH group (**D**,**E**). All data are shown as the mean ± SEM.

Since PTH1R activation was classically associated with the PKA and PKC signaling cascades to regulate target genes [23], we next assessed a possible mechanism whether IP3R-3 was modulated by PTH1R and its downstream pathway. OMSCs were treated with PKA-signaling activator (Fsk) for 24 h, finding that Fsk dramatically and steadily inhibited the level of Itpr3 (Figure 5A). On the contrary, the expression of Itpr3 was diminished using PKC activator (PMA) within 30 min, while recovered in a prolonged exposure (Figure 5B). Western blot pointed PKA signaling downregulated IP3R-3′s protein concentration (Figure 5C). Furthermore, when pre-incubating OMSCs with an inhibitor of PKA (H89) or PKC (Bis I) for 2 h prior to stimulation of PTH for 10 min to active PTH1R signaling, we observed that it was increased in both transcriptional and translational levels of IP3R-3 during PKA pathway inhibition instead of PKC pathway inhibition (Figure 5D,E). These data suggest that PTH1R activation dampened IP3R-3 expression mainly via PKA pathway (Figure 5F).

### 2.6. IP3R-3 Diminished Osteogenesis Based on Pro-Apoptosis Effect

**Figure 6:**
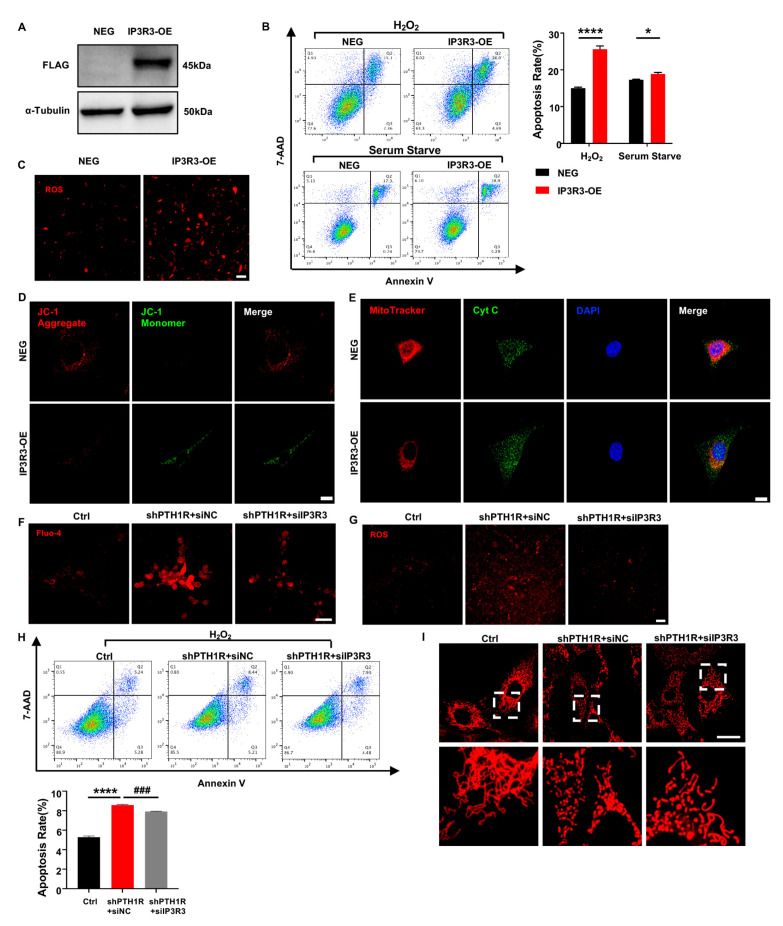
IP3R-3 plays a critical role when PTH1R is silenced. (**A**) Western blot identified the transfection efficiency which labeled with FLAG. n = 3. (**B**) Annexin V/7-AAD assay and quantitative analyses in OMSCs with IP3R-3 overexpression (IP3R3-OE) and vehicle control (NEG). n = 6. (**C**) Representative pictures displayed ROS levels in IP3R3-OE and NEG groups. n = 3. Scale bar: 100 μm. (**D**) JC-1 staining found an abnormal mitochondrial outer membrane potential (MOMP) in IP3R3-OE. n = 3. Scale bar: 25 μm. (**E**) Double fluorescence staining of Cyt C and MitoTracker analyzed the distribution of Cyt C in IP3R3-OE. n = 3. Scale bar: 25 μm. (**F**) Fluo 4-AM dyeing to labeling cytoplasmic Ca^2+^ verified siIP3R3 reduces intracellular Ca^2+^ concentration in OMSCs. n = 3. Scale bar: 50 μm. (**G**) ROS assay kit measured ROS levels in alive OMSCs with both PTH1R and IP3R-3 silenced (shPTH1R+siIP3R3), compared with PTH1R single ablated (shPTH1R+siNC) and negative control (Ctrl). n = 3. Scale bar: 100 μm. (**H**) Annexin V/7-AAD analyses in three groups. n = 5. (**I**) MitoTracker staining in alive OMSCs. n = 3. Scale bar: 25 μm. * *p* < 0.05, **** *p* < 0.0001 was considered as a statistically significant difference between NEG and IP3R3-OE or Ctrl and shPTH1R+siNC, **^###^** *p* < 0.001 was considered as a statistically significant difference between shPTH1R+siNC and shPTH1R+siIP3R3. All data are shown as the mean ± SEM.

To understand the effect of IP3R-3 in alveolar bone formation, lentivirus transfection was conducted to overexpress IP3R-3 in OMSCs (IP3R3-OE), and western blot verified the transfection efficiency (Figure 6A). After that, cell biological behaviors of IP3R3-OE were investigated. OMSCs were induced by H_2_O_2_ for 24 h or cultured in serum-free medium for 72 h, and apoptotic response was analyzed. As expected, the results exhibited that there was a slightly higher population of inchoate apoptosis in IP3R3-OE group in serum-starved medium and H_2_O_2_-aggravated apoptosis of IP3R3-OE (Figure 6B). Moreover, mitochondria were dysfunctional when IP3R-3 was overexpressed, as demonstrated by an increase in cytoplasmic ROS, JC-1 monomer, and Cyt C protein of in cytoplasm, accompanied by an imbalance in mitochondrial morphology dynamics in IP3R3-OE (Figure 6C–E). These results indicat that the IP3R-3 overexpression accelerated mitochondrial-associated apoptosis, in line with the above findings in PTH1R-knockdown OMSCs.

To assess the role of IP3R-3 in aberrant apoptosis, siRNA was executed to downregulated expression of IP3R-3 in shPTH1R. We first observed a significant reduction of intracellular Ca^2+^ when IP3R-3 was diminished (shPTH1R+siIP3R3 group) (Figure 6F), and a limited fluorescence intensity of ROS was exhibited in cytoplasm of shPTH1R+siIP3R3 group (Figure 6G). Moreover, silencing the IP3R-3 counteracted the excess percentage of inchoate apoptosis induced by PTH1R knockdown (Figure 6H). MitoTraker staining showed that the number of mitochondria increased in the shPTH1R+siIP3R3 group, and fragmented mitochondria were reversed into elongated tubular mitochondria in the shPTH1R+siIP3R3 group (Figure 6I). These data support the hypothesis that IP3R-3 plays a nonredundant role in aberrant apoptosis in PTH1R-deleted OMSCs.

**Figure 7:**
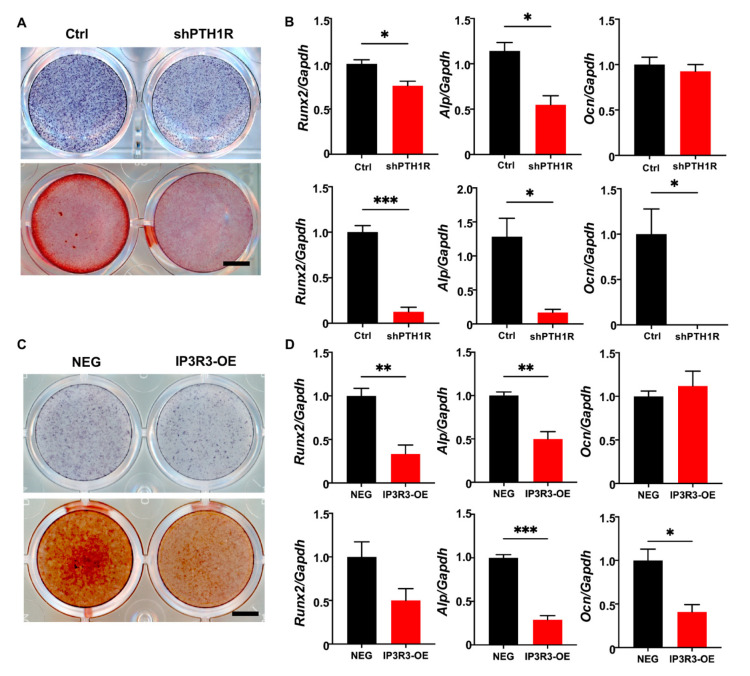
OMSCs with IP3R-3 overexpression exhibited a limited osteogenic potential, which is same as PTH1R deficiency. (**A**) Alkaline phosphatase (ALP) staining and alizarin red staining (ARS), respectively, at 7 and 14 days (d) of osteogenic induction in shPTH1R and Ctrl. n = 3. Scale bar: 5 mm. (**B**) Osteogenic related genes expression of Alp, Runx2, Ocn at 7 d (up) and 14 d (down). n = 3. (**C**,**D**) ALP and ARS staining in IP3R3-OE and NEG groups at 7 d and 14 d, and osteogenic-related genes expression were detected at 7 d (up) and 14 d (down). n = 3. Scale bar: 5 mm. * *p* < 0.05, ** *p* < 0.01, *** *p* < 0.001 was considered as a statistically significant difference between Ctrl and shPTH1R (**B**) or NEG and IP3R3-OE (**D**). All data are shown as the mean ± SEM.

It was well-established that PTH1R deletion decreased bone formation because of a diminished osteogenic ability of the osteoblast lineage cells. We first verified mineralization capacity in vitro during PTH1R knockout, which was in line with our previous findings (Figure 7A,B). Furthermore, IP3R3-OE showed a weak osteogenic ability, similar to PTH1R-deficient OMSCs, after being induced in osteogenic medium for 7 or 14 days. To be specific, we found reduced intensities of alkaline phosphatase (ALP) and alizarin red S (ARS) staining in IP3R3-OE (Figure 7C). Meanwhile, the expression of osteogenic markers was reduced in IP3R3-OE, suggesting that OMSCs with IP3R-3 overexpression recapitulates the phenotypes of PTH1R mutant (Figure 7D).

## 3. Discussion

Herein, we have demonstrated that PTH1R deletion in Prx1^+^-mesenchymal cells resulted in a decreased alveolar bone volume due in part to cellular apoptotic response activation. We have also showed that PTH1R signaling modulated the apoptosis of OMSCs, and mitochondria participated in this regulation. Importantly, IP3R-3, a potential target gene of PTH1R/PKA pathway, was essential for OMSCs fate via intracellular Ca^2+^ transport. Our results have elucidated the mechanisms of PTH1R signaling inhibiting apoptosis, highlighting that aberrant apoptosis of OMSCs disturbs mandibular development.

OMSCs belong to a special group of MSCs that reside in craniofacial bone marrow. They can differentiate into osteoblast lineage cells to speed up bone formation. Different from long bone-derived MSCs, OMSCs are sourced from migrating cranial neural crest cells and exhibit promising potential for regenerative medicine owing to their higher proliferation and mineralization ability [24]. With the development of the transgenic technique, multiple markers of MSCs in craniofacial bone have been identified via lineage-tracing analyses and lineage-ablation analyses, including *Gli1*, *Axin2*, *Prx1*, *Sp7*, and *Lepr* [25,26,27]. *Prx1* is used as a marker to define the skeletal stem cell populations. It has been proved that Prx1^+^ cells reside in the calvarial suture niche and contribute to mandibular and calvarial bone defect regeneration in 6–8-week-old mice [28,29]. We have previously demonstrated that Prx1^+^ cells were a subset of craniofacial mesenchyme and located at the base of molars and around the incisors in the mandible [21]. Thus, in this study, we focused on the pathologic changes of the mandibular alveolar bone when PTH1R was specifically deleted in the Prx1^+^ mesenchymal cells, and explored the impact of PTH1R signaling on OMSCs. Since Prx1^+^ mesenchymal cells were a subpopulation of OMSCs, lentivirus was performed to efficiently knock down PTH1R expression in OMSCs in vitro.

In the oral and maxillofacial regions, PTH1R is extensively expressed in osteoblast lineage cells, cementoblasts, and periodontal ligament cells [30]. One clue is that mice with PTH1R ablation in Sp7^+^ progenitor cells exhibited significantly truncated roots lacking periodontal ligaments due to irregular cementogenic differentiation and the impaired proliferation ability of osteoprogenitors [13]. In accordance with this research, we observed that the deletion of PTH1R in Prx1^+^ mesenchymal cells contributed to the lower alveolar bone mass, which may be caused by restrained osteogenic differentiation. Moreover, we found an active apoptotic response in the alveolar bone marrow of mutant mice, along with no difference in cellular proliferation. In fact, the skeleton is inactive at the cellular level, and cellular apoptosis is required in physiology and pathology. Mediated via a cascade of molecular events, apoptosis orchestrates the balance between cell proliferation and cell death in bone marrow [31]. Inappropriate survival and apoptosis of bone cells, which leads to interruption in bone remodeling, are the characteristics of some bone diseases, such as osteoporosis and malignant osteolysis [32]. Therefore, we suspected that apoptosis may, to a certain extent, occupy a considerable position for alveolar bone aberration during PTH1R deletion. Some works reported a suppression of apoptosis in mature osteoblasts and osteocytes during PTH treatment [33,34], while others found that PTH treatment promoted apoptosis in human embryonic kidney 293 cells and hypertrophic chondrocytes [35]. The conflicting effects of PTH1R signaling may depend on cell types and lineages. Our data suggest that PTH1R signaling play anti-apoptotic effect in OMSCs. First, we observed an increasing apoptotic population distribution in alveolar bone marrow rather than in tooth-related structures when PTH1R was specifically deleted in Prx1^+^ mesenchymal cells. Second, PTH1R knockout in OMSCs led to active apoptotic progress in vitro, concomitant with a striking mitochondrial abnormality.

Apoptosis is a cellular auto-destructive process that is precisely controlled by a plethora of factors. Among them, mitochondria play a crucial role in the apoptotic pathway by inducing caspase activation and the release of small pro-apoptotic molecules. Accumulated intracellular ROS is associated with mitochondrial dysfunction. With the development of detection methods, such as the application of scanning electrochemical microscopy, a recent study found that both cell viability and the cultivated condition affect the redox potential of MSCs. Though we found ROS accumulation in PTH1R-deleted OMSCs in vitro, the ROS levels in mandibular tissue are necessary to investigate in the future [36]. Furthermore, our study reported that PTH1R-ablated OMSCs suffered from a collapse of mitochondrial membrane potential and disruption of mitochondrial fusion, which were considered irreversible points of death cascade. Another interesting finding is that a closer spatial distance between ER and mitochondria was observed by TEM. Physiologically, ER and mitochondria are interconnected, and the release of Ca^2+^ from ER is rapidly taken up by adjacent mitochondria. Ca^2+^ transfer from ER to mitochondria is recognized as a modulator of mitochondrial function. Transient Ca^2+^ release promotes mitochondrial ATP production. However, when massive Ca^2+^ accumulates in the mitochondria, Ca^2+^ interacts with cyclophilin D, which leads to the opening of the mitochondrial permeability transition pore and the release of Cyt C [37]. In our study, an accumulation of fluorescent Fluo 4-AM Ca^2+^ indicators in mutant OMSCs further verified a close relationship between ER and mitochondria.

ER harbors different kinds of channels, pumps, and exchangers for intracellular Ca^2+^ transfer. Specific ER-to-mitochondria Ca^2+^ flux is highly dependent on the Ca^2+^-release channel IP3Rs in ER, which contributes to the physical link at the close apposition of membranes of ER and mitochondria [38,39]. Notably, three IP3R isoforms facilitate different Ca^2+^ dynamics and biological functions. IP3R-1 and IP3R-3 are of great importance in the context of Ca^2+^-associated apoptosis [40]. This may explain our observation that the expressions of IP3R-1 and IP3R-3 were enhanced in the mandible of PTH1R-ablated mice while IP3R-2 expression had no significant change. Furthermore, although IP3R-3 is not the predominant isoform, some evidence suggests an enrichment of IP3R-3 at mitochondria-associated ER membranes [41]. Thus, IP3R-3 is considered to play a preferential role in the transmission of the Ca^2+^ signal. Immunologic data demonstrated that the IP3R-3 isoform is widely expressed in the mandible, and the limitation of PTH1R signaling augmented IP3R-3’s expression. Subsequently, blocking the expression of IP3R-3 in PTH1R-deleted OMSCs rescued an excess percentage of apoptosis and disruption of mitochondrial morphology, emphasizing the necessity of IP3R-3 in the anti-apoptotic effect of PTH1R signaling. Nowadays, many researchers are interested in the controversial function of IP3R-3 in the cancer field. Specifically, a high expression level of IP3R-3 has been recognized in various cell lines and kinds of epithelial cancers, whereas other literature regards upregulation of IP3R-3 expression as a promising strategy to suppress oncogenesis [20,42]. These opposing effects are mainly due to the intracellular Ca^2+^ signal. Ca^2+^ is well-characterized as a modulator in multiple biological pathways and the function of IP3R-3-mediated Ca^2+^ transfer is complex [43]. Although a moderate concentration of Ca^2+^ in mitochondria promotes anabolic metabolism and cellular proliferation, mitochondrial Ca^2+^ overload accelerates the apoptosis process [44]. Herein, we originally discovered that overexpression of IP3R-3 in OMSCs activates the apoptotic response and promotes mitochondrial dysfunction. This observation is in line with an oncogenic feature of IP3R-3 degradation in a colorectal carcinoma model [45]. Thus, we speculated that IP3R-3 mediates apoptotic sensitivity of OMSCs during PTH1R deletion.

Furthermore, we also initially dissected the mechanism of the rising expression of IP3R-3. Since the PKA and PKC pathways are activated via Gq and Gs proteins when PTH1R binds with its ligands [46], their activators and inhibitors were used to mimic partial impact of PTH1R signaling. Both PKA and PKC pathway activation inhibited the expression of IP3R-3 in a short time, but the PKA pathway exhibited a more sustained effect. It gives detailed insights into the mechanism by which PTH1R signaling inhibits IP3R-3 transcription via the PKA pathway.

Despite uncovering a novel regulatory pattern of PTH1R signaling in cellular apoptosis, there are some limitations in our study. First, it should be noted that differences in Ca^2+^ patterns, such as calcium oscillations and calcium spikes, deliver numerous messages. More evidence is needed for the association of Ca^2+^ patterns and PTH1R signaling. Second, apoptosis belongs to a type of programmed cell death (PCD), which depends on a cascade of cytokines with tight structures. Although apoptosis is extensively studied in bone metabolism, other events have also been observed in the bone marrow cells, such as autophagy, necroptosis, and ferroptosis. The importance of PCD in bone remodeling warrants further investigation. Moreover, although we originally discovered that OMSCs with IP3R-3 overexpression decreased osteogenic differentiation ability, a histological exploration of IP3R-3 overexpression in vivo would provide stronger support.

## 4. Materials and Methods

### 4.1. Animals

All animal experiments were carried out in accordance with the guidelines of the Institutional Animal Care and Use Committee at the State Key Laboratory of Oral Diseases, Sichuan University (WCHSIRB-S-2021-339), and the Institutional Animal Care and Use Committee at the Sun Yat-Sen University (SYSU-IACUC-2022-000468). Mice with a PTH1R conditional knockout were described previously based on the Cre-LoxP recombination system [21]. Specifically, PTH1R^fl/fl^ mice were hybridized with Prx1Cre mice to generate mice lacking PTH1R in progenitors. Genomic DNA was isolated from tails and was used for genotyping. Wild-type mice (C57BL/6J) were purchased from Chengdu Dossy Biological Technology Co., Ltd (Chengdu, China).

### 4.2. Histologic and Immunologic Staining

Mandible tissues were dissected and fixed in 4% paraformaldehyde overnight and then stored in 70% ethanol at 4 °C before processing. Decalcified in 20% EDTA (pH 7.5) for one month, the samples were embedded in paraffin and then cut into 5 μm sections using an HM360 microtome (Thermo fisher Scientific, Waltham, MA, USA). Hematoxylin and eosin (H&E, Biosharp, Hefei, China), Masson’s trichrome (Solarbio, Beijing, China), and Von kossa (Solarbio) staining were performed using the manufacturers’ protocols for morphological observation. TUNEL can label the deoxynucleotidyl transferase dUTP nick end to detect apoptosis cells. TUNEL assay was performed by in situ Cell Death Detection Kit (Roche Diagnostic, Rotkreuz, Switzerland) based on instructions. Immunohistochemical (IHC) staining was performed by an Anti-Mouse ABC Staining Kit (Vector Laboratories, Newark, CA, USA), following the manufacturer′s instruction and an antibody of cathepsin K (1:100, Santa Cruz Biotechnology, Ilhéus, BA, Brazil). For immunofluorescent (IF) staining, the slides were incubated with anti-phospho-Histone H2A.X (1:400, Cell Signaling Technology, Danvers, MA, USA, #9718) and anti-IP3R3 (1:200, BD Biosciences, San Jose, CA, USA, 610312) overnight at 4 °C and stained with Alexa Fluor 568 or 488 (1:1000, Invitrogen, Carlsbad, CA, USA, A10042, A11070) for 1 h at room temperature, respectively. DAPI (Vector Laboratories) was used for counterstaining. Images of TUNEL and IF staining were captured by Olympus confocal microscope FV3000 (Olympus Inc., Tokyo, Japan). Quantitative analysis was conducted by a blinded observer.

### 4.3. Cell Culture

OMSCs were isolated from mandibular bone marrow using enzymic digestion. After removing the attached soft tissues, incisors and molars were extracted clearly and condylar cartilages were removed under stereomicroscope (Leica Biosystems Inc., Buffalo Grove, IL, USA). Alveolar bone was cut into pieces and digested by 3 mg/mL collagenase type I (Worthington Biochem, Lakewood, NJ, USA) and 4 g/mL dispase II (Roche Diagnostic) for 60 min at 37 °C. Then, cells were obtained through 70 μm cell strainers (Corning, Somerville, MA, USA) and seeded on a 100-mm dish (Corning). The cells were cultured in α-MEM (Gibco, Waltham, MA, USA) with 1% penicillin-streptomycin (HyClone, Logan, Utah, USA) and 10% fetal bovine serum (FBS) (Gibco). Trypsin (HyClone) digested cells when they reached over 80% confluence.

For osteogenic induction, OMSCs were seeded at 12-well plates with 80% confluence and cultured in osteogenic medium (10 mM β-glycerophosphate and 50 μg/mL ascorbic acid). Alkaline phosphatase staining (Beyotime, Shanghai, China) was performed on the 7th day, and alizarin red S staining (Cyagen, Suzhou, China) was executed after 14 days of induction.

To explore a possible mechanism, activators targeting protein kinase A (PKA) (FSK, MCE, 40 μM) and protein kinase C (PKC) (PMA, MCE, 10 μM) were used to mimic partial effects of PTH1R signaling activation, and inhibitors of PKA (H89, MCE, 10 μM), and PKC (Bis I, MCE, 5 μM) were added into the normal culture system 2 h prior to PTH stimulation (Teriparatide, MCE, 100 nM).

### 4.4. RNA Interference and Overexpression

For lentiviral transfection, Supplementary Table S1 shows all shRNA targeting sequences which were used to knock down PTH1R. Targeting sequences were cloned into the pHBLV-U6-MCS-PGK-PURO vector, and shPTH1R lentivirus was generated by Hanbio. Moreover, IP3R-3 overexpressed lentivirus was constructed by GeneCopoeia (Guangzhou, China). Lentiviral transfer vector (pEZ-Lv156) was first generated and purified. Restriction enzymes were used to insert the target DNA sequence binding with the Flag label. On the other hand, double-stranded siRNA vectors, which were specific for IP3R3, were designed by Hanbio (GCAGCUAGACAGAAGCAAA TT and UUUGCUUCUGUCUAGCUGC TT). OMSCs were transfected with siIP3R3 by Lipofectamine Stem Transfection Reagent (Invitrogen).

### 4.5. Annexin V-FITC and 7-AAD Flow Cytometry

The percentage of apoptosis cells was assessed after H_2_O_2_ treated for 24 h or in serum-free medium for 72 h by an Annexin V-FITC/7-Amino Actinomycin D (7-AAD) apoptosis kit (Elabscience, Wuhan, China). Cells were seeded in a 60 mm dish and incubated until they reached 80% confluence. Afterwards, floating and adherent cells were collected and resuspended in Annexin V binding buffer (approximately 106 cells per 100 μL). 2.5 μL FITC-labeled Annexin V and 2.5 μL 7-AAD were added into 100 μL cellular suspension and incubated for 20 min at room temperature in the dark. Diluted with 400 μL Annexin V binding buffer, flow cytometry was performed (Backman Coulter, Brea, CA, USA).

### 4.6. Determination of Mitochondrial Outer Membrane Potential (MOMP)

The lipophilic cationic fluorescent dye JC-1 (Beyotime) was used to assess the MOMP in OMSCs. The cells were seeded into the confocal dishes with 4 × 10^4^ cells/well for 24 h. Then, the medium was discarded and the cells were incubated in 1 mL fresh medium with 2 μL JC-1 staining fluid for 30 min at 37 °C in the dark. Washed twice by PBS, the cells were observed with the Olympus confocal microscope FV3000 (Olympus).

### 4.7. MitoTracker and Immunocytochemistry (ICC) Staining

For analysis of mitochondrial morphology, a mitochondrial potential-independent dye, MitoTracker Deep Red FM (Invitrogen), was preformed, based on instruction. Briefly, cells were sowed into 8-well slides with 4 × 10^4^ cells/well and cultured for 24 h. Discarded with medium, the cells were cultured in growth medium (serum free) with 500 nM dye for 45 min at 37 °C in the dark. After the staining was complete, the cells were directly observed by a confocal microscope (Olympus) or were sequentially performed with ICC staining. Anti-cytochrome c (Cyt C, 1:200, BD Biosciences, 556433) or IP3R3 (1:200, BD Biosciences, 610312) was incubated for immunostaining.

### 4.8. Transmission Electron Microscope (TEM)

The OMSCs were fixed, dehydrated, and finally embedded. Then ultrathin sections were cut with diamond knife and the semithin sections were stained with uranyl acetate and citrate. The sections were examined with JEM-1400-FLASH Transmission Electron Microscope and micrographs (JEOL Ltd., Akishima City, Tokyo, Japan) were collected. ImageJ2 was used to analyze the distance between the mitochondrial outer membrane and the ER membrane, and the interface lengths of two organelles with 100 nm gap distance.

### 4.9. Measurement of Intracellular Ca^2+^ Concentration

Intracellular Ca^2+^ concentration was revealed with Fluo-4 AM vial (Invitrogen). The cells were cultured until they reached over 90% confluence and incubated with Flou-4 AM in the dark for 1 h. Then, the OMSCs were washed twice with DPBS and incubated in washing buffer for an additional 30 min at room temperature. The changes of Ca^2+^ concentrations were monitored by 494-nm light from a flow cytometer (Backman Coulter) or a confocal microscope (Olympus).

### 4.10. RNA Extraction and Quantitative Real-Time PCR (qRT-PCR)

The total RNA from mice mandible at P21 was extracted by Trizol (Invitrogen) according to the instruction, and the cellular RNA was executed using the Takara Minibest Universal RNA Extraction Kit (Takara, Kusatsu, Shiga, Japan). The concentration of RNA was determined with the absorbance (NanoDrop ND-1000, Thermo Fisher Scientific). cDNA was reversed by RNA using the PrimeScript RT Reagent Kit (Takara) and the expression was evaluated by LightCycler^®^ 480 II (Roche) with SybrGreen Supermix (Bio-Rad Laboratories, Hercules, CA, USA). Gapdh was measured for normalization. The primers are listed in Supplementary Table S2.

### 4.11. Protein Extraction and Western Blot

Proteins from cells were extracted by whole-cell lysis assay (KeyGEN, Nanjing, Jiangsu, China) and measured by SpectraMax^®^ iD3 (Molecular Devices, CA, USA) using an enhanced BCA protein assay kit (Beyotime). Equal amount of protein samples were separated by NuPAGE 4–12% Bis-Tris Gel (Invitrogen) and transferred onto a nitrocellulose blotting membrane (GE Healthcare Life Sciences, Pittsburgh, PA, USA). Blocking with 5% BSA for 1 h, the membrane was incubated with anti-IP3R3 (1:2000, BD Biosciences, 610312), FLAG (1:1000, Santa Cruz, sc-166355) and β-actin (1:1000, SAB, 41517) overnight. Incubated with Goat anti-rabbit or mouse IgG secondary antibody HRP conjugated (1:5000, Signalway Antibody, Sioux Falls, South Dakota, USA, L3012/L3032) for 1 h at the following day, the membrane was probed by an enhanced chemiluminescence kit (Bio-Rad Laboratories).

### 4.12. Statistics

All experiments were performed independently in triplicate. GraphPad Prism 9.0 was used for statistical analysis. Two-group comparisons were analyzed by unpaired two-tailed Student’s *t*-test, and multiple comparisons were evaluated by one-way analysis of variance (ANOVA) with a Bonferroni post hoc test. All data were expressed as mean ± SEM. The level of significance was set at *p* values < 0.05.

## 5. Conclusions

In conclusion, we have demonstrated the anti-apoptotic regulation of PTH1R in OMSCs, as well as the contribution of cellular apoptosis to osteogenesis. Our evidence also supports that IP3R-3 is at the core of PTH1R signaling-associated apoptosis, and PTH1R and its downstream PKA pathway dampen IP3R-3′s expression. These findings may provide new insights into the effects of PTH1R on mandibular growth.

## Data Availability

Data are contained within the article.

## Supplementary Materials

The following supporting information can be downloaded at: https://www.mdpi.com/article/10.3390/ijms252312607/s1.
