# Supplementary material for: PTH1R Suppressed Apoptosis of Mesenchymal Progenitors in Mandibular Growth
Source: Int J Mol Sci. 2024 Nov 24;25(23):12607. doi: 10.3390/ijms252312607 (PMC11641607; doi:10.3390/ijms252312607)
Supplement: Supplementary file 1 [file ijms-25-12607-s001.zip › ijms-3302210-supplementary.pdf]

PTH1R Suppressed Apoptosis of Mesenchymal Progenitors in Mandibular Growth

Chen Cui [1,2,†], Chuang Lu [1,2,†], Yanling Cai [1,2], Yuhua Xiong [1,2], Yihong Duan [1,2], Kaiwen Lan [1,2], Yi Fan [3], Xuedong Zhou [3,*] and Xi Wei [1,2,*]

Supplementary Table S1

shRNA target sequences

Negative control (Ctrl)	TTCTCCGAACGTGTCACGTAA
shRNA1	CAGCCAACATAATGGAGTCAGACAA
shRNA2	ACACTGGCATTGGACTTCAAGCGTA
shRNA3	CACCTCCATTGTTGCAGGAAGAATG

Supplementary Table S2

qRT-PCR primer pairs

	Forward primer	Reverse primer
Caspase 8	TGCTTGGACTACATCCCACAC	TGCAGTCTAGGAAGTTGACCA
Caspase 9	GACGCTCTGCTGAGTCGAG	GGTCTAGGGGTTTAACAGCCTC
Caspase 3	TGGTGATGAAGGGGTCATTTATG	TTCGGCTTTCCAGTCAGACTC
Caspase 6	GGAAGTGTTCGATCCAGCCG	GGAGGGTCAGGTGCCAAAAG
Caspase 7	AAGACGGAGTTGACGCCAAG	CCGCAGAGGCATTTCTCTTC
PARP	GGCAGCCTGATGTTGAGGT	GCGTACTCCGCTAAAAAGTCAC
Apaf1	AGTGGCAAGGACACAGATGG	GGCTTCCGCAGCTAACACA
Bcl2	GTCGCTACCGTCGTGACTTC	CAGACATGCACCTACCCAGC
PTH1R	TTTCCCGGTGCCTTCTCTTTC	CAGGCGCAATGTGACAAGC
IP3R-1	CGTTTTGAGTTTGAAGGCGTTT	CATCTTGCGCCAATTCCCG
IP3R-2	TTCAGTTCCTATCGAGAGGATGT	GCTGATTGACGCAAGGTCG
IP3R-3	GGGCGCAGAACAACGAGAT	GAAGTTTTGCAGGTCACGGTT
Alp	CACGGCCATCCTATATGGTAA	GGGCCTGGTAGTTGTTGTGA
Runx2	TCCACAAGGACAGAGTCAGATTA CAG	CAGAAGTCAGAGGTGGCAGTGT CATC
Ocn	CTGACCTCACAGATCCCAAGC	TGGTCTGATAGCTCGTCACAAG
Gapdh	ACTGAGGACCAGGTTGTC	TGCTGTAGCCGTATTCATTG
